# Vaccine-associated enhanced disease in humans and animal models: Lessons and challenges for vaccine development

**DOI:** 10.3389/fmicb.2022.932408

**Published:** 2022-08-10

**Authors:** Julie Bigay, Roger Le Grand, Frédéric Martinon, Pauline Maisonnasse

**Affiliations:** Immunology of Viral Infections and Autoimmune Diseases (IMVA), IDMIT Department, Institut de Biologie François-Jacob (IBJF), University Paris-Sud-INSERM U1184, CEA, Fontenay-Aux-Roses, France

**Keywords:** vaccine, vaccine-associated enhanced disease, immunology, infectious diseases, immune response

## Abstract

The fight against infectious diseases calls for the development of safe and effective vaccines that generate long-lasting protective immunity. In a few situations, vaccine-mediated immune responses may have led to exacerbated pathology upon subsequent infection with the pathogen targeted by the vaccine. Such vaccine-associated enhanced disease (VAED) has been reported, or at least suspected, in animal models, and in a few instances in humans, for vaccine candidates against the respiratory syncytial virus (RSV), measles virus (MV), dengue virus (DENV), HIV-1, simian immunodeficiency virus (SIV), feline immunodeficiency virus (FIV), severe acute respiratory syndrome coronavirus 1 (SARS-CoV-1), and the Middle East respiratory syndrome coronavirus (MERS-CoV). Although alleviated by clinical and epidemiological evidence, a number of concerns were also initially raised concerning the short- and long-term safety of vaccines against severe acute respiratory syndrome coronavirus 2 (SARS-CoV-2), which is causing the ongoing COVID-19 pandemic. Although the mechanisms leading to this phenomenon are not yet completely understood, the individual and/or collective role of antibody-dependent enhancement (ADE), complement-dependent enhancement, and cell-dependent enhancement have been highlighted. Here, we review mechanisms that may be associated with the risk of VAED, which are important to take into consideration, both in the assessment of vaccine safety and in finding ways to define models and immunization strategies that can alleviate such concerns.

## Introduction

Vaccination is considered to be one of the most-cost effective medical interventions of the 20*^th^* century (Préaud et al., 2015; Ozawa et al., 2016; Leidner et al., 2019): The significant decrease in global morbidity and mortality associated with a number of infections (Greenwood, 1645; Rappuoli et al., 2019) has synergized with the economic benefits in healthcare costs brought by the implementation of such preventive measures. Today, the goals of modern vaccinology in the context of infectious diseases are to: (A) create vaccines against (re)emerging diseases; (B) handle refractory pathogens for which efficient vaccines have proven difficult to develop, and (C) improve vaccination strategies, including vaccine accessibility (Greenwood, 1645; Centlivre and Combadière, 2015; Pollard and Bijker, 2020; Excler et al., 2021). Understanding and controlling the immune mechanisms elicited by such vaccines is crucial in moving forward in this quest. Beyond the fear of developing an ineffective vaccine, a concern (Graham, 2020) in the field of vaccinology is the formulation of vaccines that, upon subsequent natural infection, may intensify the severity and worsen the prognosis of the pathology they were designed to protect against; a process known as a vaccine-associated enhanced disease (VAED) (Huisman et al., 2009; Jamrozik et al., 2021a; Munoz et al., 2021). More specifically, VAED is defined in this review as the immune-mediated aggravation of the clinical course of infection following immunization relative to that in the absence of previous vaccination. VAED varies between vaccine platforms and may be caused by distinct mechanisms that are not yet completely understood or have not been entirely explored. Furthermore, to date, no factor has been shown to consistently predict the occurrence of VAED or even differentiate it from severe natural infection. Also, it is likely that no single factor will be able to predict VAED risk, as it might be pathogen-dependent.

In this article, we review the pathophysiological mechanisms that may lead to immune-mediated exacerbation of the disease. Then, we describe the preclinical and clinical cases in which VAED has been observed with vaccine candidates against diverse human and animal pathogens. Furthermore, we address the questions that were initially raised concerning the risk of VAED with anti-SARS-CoV-2 vaccines, as well as the extent to which it should be monitored. Finally, we discuss the use of preclinical and clinical models and how, in addition to vaccine formulation, they can be improved to better address safety in vaccine development.

## Describing pathophysiological pathways suspected to lead to vaccine-associated enhanced disease

The purpose of vaccination is to induce a long-lasting immune response and/or immune memory that can be rapidly unleashed during a future encounter with the pathogen being vaccinated against (Pollard and Bijker, 2020). In certain circumstances, the immune responses triggered by exposure to antigens can, individually or through their association, favor exacerbated disease upon subsequent exposure to that same pathogen. Although this process has been described to occur naturally in the case of dengue virus (DENV) after sequential exposition to different serotypes (Halstead, 2007; Guzman et al., 2013), vaccination against most infectious diseases has resulted in only very rare cases of VAED (Huisman et al., 2009; Jamrozik et al., 2021a; Munoz et al., 2021). In the few reported cases, the enhanced disease was due to failed efforts of the immune system to control the infection and subsequently manifested with symptoms related to the organ(s) targeted by the virus. Thus, VAED generally presents as an exacerbation of the pathology seen in natural infection and addressing VAED requires a proper understanding of the course of such an infection and the host-pathogen interactions that take place.

With the exception of the anti-DENV vaccine developed by Sanofi Pasteur and the FI-MV vaccine, which were withdrawn from the market, the occurrence of VAED has always been detected during preclinical and clinical experimental phases (i.e., before licensing) (Jamrozik et al., 2021a). However, continued safety monitoring is carried out even after marketing to ensure the benefits of the vaccines. While identifying VAED is crucial for the development of future safe vaccines, we still lack a clear understanding of the exact associated mechanisms that lead to enhanced pathogenesis.

### Antibody-dependent enhancement of disease

Various vaccines use antibody titers as correlates of protection, i.e., reaching a specific antibody level following immunization is statistically associated with protection from infection and/or disease (Plotkin, 2010). Neutralizing antibodies attach to the receptor-binding domain (RBD) of proteins at the surface of viruses and prevent binding to the cellular receptor or blocking fusion mechanisms of the viral envelope with target cell membranes (Taylor et al., 2015). Neutralization follows a widely-accepted model, in which it depends on the stoichiometric threshold of neutralization, antibody affinity (and avidity), and, importantly, epitope accessibility (Pierson et al., 2008; Dowd et al., 2011). Both neutralizing and non-neutralizing antibodies can have additional antiviral properties by recruiting other components of the immune system, providing a direct link between innate and adaptive immune responses (Lu et al., 2017; Sedova et al., 2019; Van Erp et al., 2019). These additional effector functions require simultaneous binding to the target antigen on the surface of the pathogen or infected cell via their antigen-binding fragments (Fab region), which forms an immune complex, and the Fc receptor (FcR) on the surface of immune effector cells via their Fc-region (Lu et al., 2017), which triggers a cascade of effector functions. Antibody-dependent cellular cytotoxicity (ADCC) is a type of cell-mediated immunity directed against infected cells opsonized by antibodies, resulting in their lysis, and has been characterized as a potent antiviral response for a number of infections. It generally involves cytotoxic cells, such as NK cells and CD8^+^ T lymphocytes, and can be enhanced via activation of the complement system. Antibody-dependent cellular phagocytosis (ADCP) provides for the clearance of virus-infected cells by recruiting phagocytes (mainly macrophages and neutrophils) (Tay et al., 2019; Bournazos et al., 2020).

**FIGURE 1:**
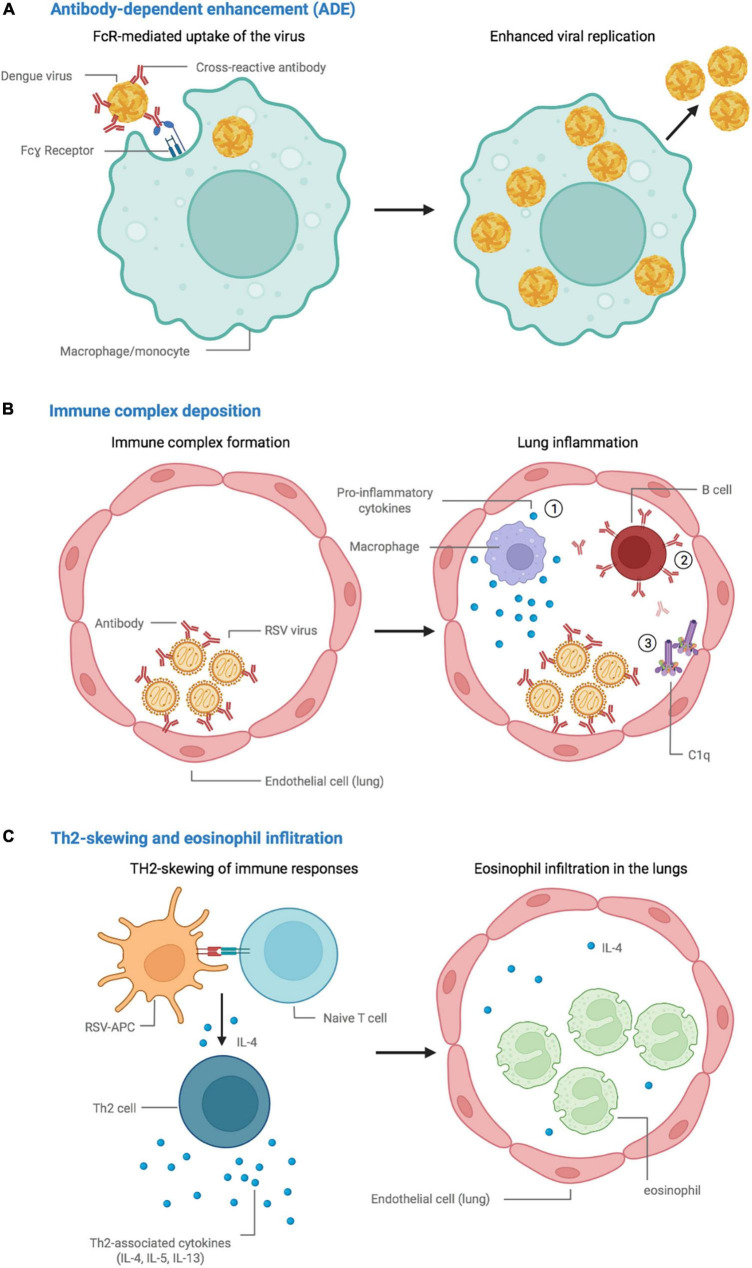
Proposed mechanisms leading to vaccine-associated enhancement of disease. **(A)** Antibody-dependent enhancement (ADE), in which cross-reactive vaccine-elicited antibodies fail to neutralize the dengue virus and instead promote viral replication in FcR-bearing cells. **(B)** Immune complex accumulation and deposition in the lungs, promote (1) an inflammatory environment, (2) the production of antibodies, and (3) activation of the complement cascade. **(C)** Skewing of the immune response toward a Th2 phenotype, followed by eosinophil infiltration and accumulation in the lungs. *Created with BioRender.com*.

Non-neutralizing, sub-neutralizing, and/or suboptimal concentrations of otherwise potent antibodies can have disproportionate Fc-mediated functions, through receptors for the Fc-region of antibodies (FcRs), which can alter immune responses (Nimmerjahn and Ravetch, 2007). First, they can, instead of blocking viral entry, lead to increased binding to and internalization into immune cells expressing FcRs, such as monocytes, macrophages, and dendritic cells (DCs) (Tirado and Yoon, 2003; Pierson et al., 2008; Dowd et al., 2011; Wang et al., 2014; Diamond and Pierson, 2015; Taylor et al., 2015; Smatti et al., 2018). In most infections, for example with coronaviruses (Tseng et al., 2005; Yilla et al., 2005; Jaume et al., 2011; Wang et al., 2020; Yang J. et al., 2020; Zheng et al., 2021; Junqueira et al., 2022), this is not necessarily indicative of ADE, as multiplication of the pathogen is mostly abortive in these cells, meaning that there is no production or dissemination of infectious particles. In the case of DENV, however, FcR-mediated uptake leads to productive replication during natural infection (Flipse et al., 2016; Wan et al., 2018; Narayan and Tripathi, 2020), a process that has been mimicked with vaccination. During infection with DENV, pre-existing cross-reactive antibodies targeting a serotype of the pathogen other than the infecting one, or even another closely related *flavivirus* (Katzelnick et al., 2021), can attach with lower affinity to the newly-infecting pathogen. A loss of neutralization capacity can ensue, as well as facilitation of infection of susceptible immune cells (Figure 1A; Halstead and O’Rourke, 1977; Halstead, 1979; Dejnirattisai et al., 2010; Katzelnick et al., 2017). This abnormal process leads to enhanced infection of susceptible cells with the pathogen, enhancing the infected cell mass, which in turn results in both an increased rate of infection and exacerbated pathology (Flipse et al., 2016). Importantly, only a small proportion of antibodies leads to ADE, whereas most contribute to overall protection conferred by ADCP (Bournazos et al., 2020). Furthermore, the accumulation and deposition of immune complexes, which have, for example, been observed in the lungs of children and animal models challenged following immunization with feline immunodeficiency-respiratory syncytial virus and -measles virus (FI-RSV and FI-MV) vaccines (Polack et al., 1999, 2002b,^1999^), can lead to overabundant Fc-mediated recruitment of immune cells, inflammation, Th2-biased responses and increase antibody production (Anderson and Mosser, 2002), subsequently enhancing the disease (Figure 1B).

Studies have also shown that another mechanism can result in persistent ADE-mediated productive infection of macrophages for prolonged time periods generated by innate immune suppression (Linn et al., 1996; Halstead et al., 2010; Ubol and Halstead, 2010). This phenomenon is called intrinsic ADE. It involves the modulation of innate immune effectors by internalized virus-immune complexes to favor increased replication and release of the virus from infected cells. In the case of ADE mediated by DENV infection, it has been shown that viral entry via FcγR-antibody complexes leads to the inhibition of pro-inflammatory cytokine production and causes a Th2 biased immune response and increased intracellular viral replication (Narayan and Tripathi, 2020).

### Complement-mediated enhancement of disease

Immune complex-mediated enhancement in the context of ADE can be further enhanced by complement receptors (CRs) which can expand the number of cells that are permissive to the virus to additional immune and non-immune cells (Takada et al., 2007). If viral replication is initiated in these target cells, the immunopathological response may be further amplified. This has been mostly reported after viral challenge in the case of VAERD for the FI-RSV) (Polack et al., 2002b,^2003^) and FI-MV vaccines, as well as DENV infection (Jane Cardosa et al., 1983; Taylor et al., 2015). This complement-mediated ADE process has also been demonstrated *in vitro* for several pathogens (Jane Cardosa et al., 1983; Robinson, 2006).

### Cell-mediated enhancement of disease

Finally, cell-mediated mechanisms have also been reported, mainly for FI-RSV and FI-MV vaccines. The preclinical and clinical evaluation suggests the initiation of vigorous CD4 T-cell responses and, in many instances, the skewing of cellular immune responses toward an apparently pathogenic phenotype. Animal models have highlighted a mechanism of Th2-mediated eosinophilic hypersensitivity responses in the lungs (Polack et al., 1999, 2002a; de Swart et al., 2002; Knudson et al., 2015) following vaccination with the FI-RSV and FI-MV vaccines (Figure 1C). Furthermore, lung eosinophilia has been linked to the absence of cytotoxic T-lymphocyte priming during vaccination, both for FI-RSV (Srikiatkhachorn and Braciale, 1997) and SARS-CoV-1 vaccines (Channappanavar et al., 2014). However, the role of eosinophils in the resulting pathogenicity is highly debated, as other studies have shown that distinct CD4^+^ T-cell responses account for specific parameters of the pathological response (Knudson et al., 2015). In the case of an FIV animal model, a mechanism of lymphocyte activation-mediated enhancement of viral replication has been characterized, in which vaccination leads to enhanced viral replication due to the recruitment of immune cells that simultaneously act as target cells for the virus (Richardson et al., 1997, 2002).

### The “original antigenic sin” phenomenon

The “original antigenic sin” phenomenon refers to the propensity of the immune system to preferentially use immunological memory built from a previous encounter with an antigen (through infection or vaccination), at the expense of protective primary responses when confronting a slightly different version of the antigen (Fazekas de St Groth and Webster, 1966; Klenerman and Zinkernagel, 1998; Kim et al., 2009). By soliciting immune memory, this process is intended to induce an efficient and rapid response (Veiga-Fernandes et al., 2000), but, instead, skews the immune system toward the first response is made (i.e., the “original antigen”). This is highly detrimental, as it prevents the construction of a potentially more effective immune response targeted against the current immunizing epitopes, which can in turn hamper the control of infection and hence lead to the exacerbation of disease (Fazekas de St Groth and Webster, 1966; Klenerman and Zinkernagel, 1998; Kim et al., 2009). This has been particularly observed in the context of the binding of non-neutralizing antibodies to non-protective epitopes, subsequently preventing the attachment of neutralizing antibodies to protective epitopes, as described for DENV infections with different serotypes (de Alwis et al., 2014; Halstead, 2014). In addition to the risk of ADE due to non-neutralizing cross-reactive antibody responses, Mongkolsapaya et al. showed ineffective cytotoxic T-cell responses that may contribute to severe Dengue disease. The recruitment of T cells with a relatively low affinity for the current infecting virus relative to likely previously-encountered strains was observed in dengue-infected Thai children (Mongkolsapaya et al., 2003), leading to impaired viral clearance and more severe disease. T-cell-mediated recall of primary responses to the detriment of *de novo* responses to a new antigen has been described for other viruses (Klenerman and Zinkernagel, 1998). Although initial observations were not confirmed in subsequent studies, in the context of coronaviruses, it was suggested that previous exposure to non-pandemic seasonal coronaviruses (HKU1, NL63, OC43, and 229E) that cause common cold-like symptoms or other pandemic coronaviruses (SARS-CoV-1, MERS-CoV) may lead to the exacerbation of COVID-19 upon subsequent exposure to SARS-CoV-2 (Focosi et al., 2021; McNaughton et al., 2022). Extension of this notion led to the early presupposed idea that COVID-19 disease might be more severe in adults than in children due to magnified exposure to coronaviruses throughout their lifespan and associated pre-existing cross-reactive non-neutralizing antibodies (Fierz and Walz, 2020). However, a number of studies suggest that cross-reactive antibodies may have a beneficial role in vaccination against coronaviruses that cause infections in humans (Tan et al., 2021).

## Vaccines and vaccine candidates with evidence of vaccine-associated enhanced disease

### Formalin-inactivated respiratory syncytial virus and measles virus vaccines

Infections with RSV are one of the main causes of acute lower respiratory infections (ALRI) in children and the elderly worldwide, with an estimated 33.1 million cases and 3.2 million hospital admissions of children under five years of age in 2015 (Shi et al., 2017). Despite the urgent need to produce a safe and effective vaccine against RSV (Shaw et al., 2013a), none are currently available on the market. An unfortunate turn of events in the United States in the 1960s (Kim et al., 1969; Smatti et al., 2018) with an FI-RSV vaccine considerably slowed subsequent research in humans. Approximately 80% of the vaccinated children in the trial had to be hospitalized because of VAED, two of whom died, which was significantly more than the 5% in the control group (Kim et al., 1969). Other than abnormal non-protective antibody responses (Murphy et al., 1986), several mechanisms are thought to have contributed to this process, namely complement deposition, the absence of cellular cytotoxic responses, and a bias toward a Th2 cellular response (Delgado and Polack, 2004). Bronchiolar pathology was observed in the hospitalized patients, and histological findings in the lungs highlighted peribronchiolar monocytic infiltration, with excessive levels of eosinophils (Kim et al., 1969). The mechanisms involved in the immunopathology following FI-RSV vaccination have been extensively studied to better understand the root causes associated with such adverse events and in the hope of producing an effective vaccine to meet the medical needs associated with this disease. In a BALB/c mouse model, FI-RSV vaccination followed by RSV challenge was associated with enhanced pulmonary eosinophilia and an increase in the total number of cells (eosinophils, granulocytes, CD4 T cells), mimicking that of children vaccinated with FI-RSV, probably due to an exaggerated Th2 memory response (Waris et al., 1996; Castilow et al., 2007). Interference with Th2 pathways, for example, depletion of IL-4 or IL-13, led to decreased pulmonary eosinophilia and lung pathology (Connors et al., 1994; Johnson and Graham, 1999; Castilow et al., 2007). Conversely, although their potential immunomodulatory role has been suggested by many studies (Hussell et al., 1997; Suzuki et al., 2002; Castilow et al., 2007), CD8^+^ T-cell numbers were shown to plummet.

The same phenomenon occurred after the licensing of an FI-MV vaccine, causing atypical measles in vaccinated individuals (Fulginiti et al., 1967; Nader et al., 1968; Martin et al., 1979; Smatti et al., 2018), characterized by fever, head, and abdominal pain, rash, and severe pneumonitis. The vaccine was removed from use in 1967, after 1.8 million people, mainly children had been vaccinated (Fulginiti et al., 1967; Martin et al., 1979). The lung histopathology observed with FI-MV was highly similar to that documented following immunization with the FI-RSV vaccine, as well as many immunological and clinical manifestations (Polack, 2007). Rhesus macaques vaccinated with FI-MV developed atypical measles, similar to that described for vaccinated infants, characterized by eosinophil infiltration and immune complex deposition in the lungs (Polack et al., 1999). As for FI-RSV, exposure to MV after FI-MV vaccination led to a Th2-biased immune response, characterized by a rapid reduction in IL-12 production and a subsequent increase in pulmonary eosinophilia and the production of IL-4 (Polack et al., 2002a).

Many studies have highlighted eosinophil infiltration as being the hallmark for VAED in animal models and humans vaccinated with FI-RSV or FI-MV and afterward infected with the target virus, but the subsequent role of eosinophil infiltration in the resulting pathology is yet to be deciphered. Experiments suggest that vaccine-induced pulmonary eosinophilia following vaccination with FI-RSV may have two roles: (1) contributing to pulmonary pathogenesis but also (2) reducing the viral load (Su et al., 2015). While IL-5 and eotaxin were shown to be mediators of leukocyte recruitment, mucus production, and, overall, FI-RSV-induced inflammation, the passive transfer of eosinophils in knockout mice accelerated viral clearance. Further evidence of the dual pathogenic pro-inflammatory and protective anti-viral role of eosinophils was provided by mouse models in response to infection with the pneumonia virus of mice (Percopo et al., 2014). Other studies somewhat exonerated the pathogenic role of eosinophils in VAED by showing that the various parameters of immunopathology associated with vaccine-enhanced RSV disease in mice are mediated by distinct CD4 T cells rather than eosinophils alone (Knudson et al., 2015).

Furthermore, vaccination with both FI-RSV and FI-MV was shown to elicit suboptimal humoral responses relative to natural infection with RSV and MV: less neutralizing antibody was elicited in vaccinated infants and children (Kim et al., 1969; Polack et al., 2002b,^2003^; Polack, 2007; Acosta et al., 2016) than those of comparable age who were naturally-infected. Before RSV infection, the vaccinated children showed an elevated ratio of binding antibodies versus neutralizing antibodies against the RSV compared to that detected in children who were naturally infected (Murphy et al., 1986). This was speculated to have contributed to the pathogenesis by delaying viral clearance. The immune complex deposition was also observed, probably favored by the presence of large amounts of non-neutralizing antibodies (Polack et al., 2002b,^2003^). Poor stimulation of T cells and subsequent improper affinity maturation of B cells in germinal centers has been postulated. In addition, in animal models, the complement cascade has been shown to play an important role in VAED following FI-RSV immunization and further RSV challenge (Polack et al., 2002b; Melendi et al., 2007). In younger children, the observed immunopathology may be associated with the immaturity of their immune system characterized by weak stimulation of TLRs (Delgado et al., 2009) and less prominent Th1 responses able to tackle viral infections (Bottazzi et al., 2020). Furthermore, the pathological immune responses leading to atypical measles and VAED following immunization with FI-MV and FI-RSV, respectively, could also be due to formalin-associated disruption of critical epitopes (Murphy et al., 1986; Castilow et al., 2007). Indeed, the generation of carbonyl groups during formalin-mediated inactivation (Castilow et al., 2007) may be a contributing factor, as pulmonary eosinophilia and Th2-associated cytokines were shown to diminish when carbonyl groups were reduced (Moghaddam et al., 2006). However, VAED was not described for the formalin-inactivated vaccines against parainfluenza (Kim et al., 1969), polio (Melnick, 1996), or hepatitis A (Werzberger et al., 1992). Thus, formalin-mediated inactivation cannot be the sole mechanism involved. Lastly, it was also established that, contrary to native RSV, which presents the F glycoprotein in its pre-fusion and post-fusion form, the FI-RSV vaccine primarily presents it in the post-fusion conformation (Killikelly et al., 2016). Hence, a vaccine with the protein stabilized in the pre-fusion state through a limited number of mutations was able to elicit high levels of neutralizing antibodies and protect cotton rats against RSV challenge (Krarup et al., 2015). A number of clinical trials are currently being held to test the immunogenicity, safety, and protective efficiency of various vaccine formulations with this pre-F protein (Beran et al., 2018; DeVincenzo et al., 2019).

### Dengue virus and vaccines

Dengue fever is a mosquito-borne disease caused by the dengue virus (DENV), a member of the *Flavivirus* genus. There are four different serotypes of the virus (DENV 1-4), each able to cause an acute systemic viral disease with a range of severity, going from mild to death by shock. With an estimated 390 million cases a year (Bhatt et al., 2013), subtropical and tropical regions are the most highly affected. Increasing travel and trade, as well as increased urbanization, are gradually increasing the territorial reach of the mosquito and hence the virus (Bhatt et al., 2013; Diamond and Pierson, 2015), resulting in massive public health and economic burden. Dengue has been extensively investigated in the context of ADE. During natural infection, antibodies induced by a first infection are generally highly neutralizing for the specific DENV serotype, thus preventing re-infection with the homologous strain in most cases. However, they appear to have poor avidity and less neutralizing capacity against heterologous DENV strains. Such natural pre-existing immunity is the main risk factor associated with severe diseases, namely dengue hemorrhagic fever (DHF) and dengue shock syndrome (DSS) (Halstead, 2007; Guzman et al., 2013).

In the 1960s, severe disease (DHF/DSS) was reported from clinical and physiological studies to be the result of a second infection by one of the other four types of DENV (reviewed in Halstead and Cohen (2015)). Studies with the rhesus macaque animal model have shown that ADE of DENV infections was observed in the presence of anti-DENV antibodies, whether actively raised (Halstead et al., 1973) or passively acquired (Halstead, 1979). *In vitro*, monocytes were cultured in the presence of DENV and the addition of anti-DENV antibodies resulted in enhanced infections (Halstead and O’Rourke, 1977). Finally, enhanced viremia during secondary dengue infections in monotypic immune humans was shown to be statistically associated with severe disease (Vaughn et al., 2000).

Studies in tissue culture have demonstrated that cross-reactive antibodies targeting the DENV envelope glycoproteins that either does not neutralize a DENV serotype or do not reach an adequate concentration to effect potent neutralization can lead to increased infection of host target cells via a mechanism based on increased binding and internalization of antibody-coated infectious virions by Fc receptors (Halstead, 2014; Gan et al., 2017).

Although severe disease associated with secondary infection with a heterologous strain is quite infrequent, it becomes a major problem upon primary infection of infants born from DENV-immune mothers whose DENV-specific IgG has crossed the placental barrier (Halstead et al., 2002; Diamond and Pierson, 2015). In this process, cross-reactive antibodies may augment the entry of the virus into cells in an FcγR-mediated fashion (Moi et al., 2011; Taylor et al., 2015), thus increasing viremia and exacerbating the severity of the disease in these children (Halstead, 2007; Diamond and Pierson, 2015). Complement receptors have also been shown to be involved in enhancing viral replication in a larger spectrum of cells through complement-dependent-ADE (Jane Cardosa et al., 1983; Taylor et al., 2015). These immune interactions have been recently extended to the closely related Zika virus (ZIKV). The risk of symptomatic DENV2 infection and severe disease is increased by prior ZIKV infection in children (Katzelnick et al., 2020a,b).

As for natural infection, vaccine-elicited antibodies against a single serotype can elicit cross-reactive antibodies that could become harmful if there is a subsequent infection with another serotype. Given the risks associated with pre-existing cross-reactive antibodies, it appears quite obvious that developing a safe vaccine against dengue involves the induction of protective antibodies against all four serotypes with equivalent efficacy. With this in mind, many strategies to develop effective and safe vaccines against DENV have been initiated (Durbin and Whitehead, 2011). The live-attenuated chimeric yellow fever tetravalent dengue vaccine (CYD-TDV or Dengvaxia) from Sanofi Pasteur was licensed in 2015 and has received endorsement by the WHO for targeted use in DENV-endemic countries. The phase III clinical trial demonstrated moderate efficacy for symptomatic dengue depending on the serotype (pooled efficacy of 60.3% 25 months after the third dose of vaccine across the various clinical trials conducted in endemic areas). The vaccine efficacy against hospitalization due to severe dengue forms (DSS or DHF) was much higher, reaching approximately 80% (Hadinegoro et al., 2015; Villar et al., 2015). However, the vaccine faced a number of issues, with increased rates of hospitalization of vaccinated seronegative children relative to the control group. Although ADE was speculated, it was shown that the sero-status of vaccinated individuals was critical: individuals that had no previous immunity to DENV had higher rates of hospitalization following infection with DENV than seropositive individuals. The WHO thus recommended using the vaccine in seropositive individuals between 9 and 45 years of age, for whom exacerbation of the disease was less observed (Halstead, 2018). In several countries, the vaccine is provided together with a serological test. A more recent re-evaluation of the results of phase III clinical trial (Sridhar et al., 2018) reinforced the hypothesis that, in the absence of previous dengue exposure, the CYD-TDV vaccine partially mimics primary infection. However, the increased risk of severe dengue during subsequent infection is still highly debated (Halstead et al., 2020; Salje et al., 2021). As for natural infection, the vaccine induces cross-reactive but non-neutralizing antibodies (Taylor et al., 2015). Potential mechanisms have been deciphered, in which antibodies may enhance cellular infection by increasing the fusion potential of the virus in macrophages, or, on the contrary, by increasing the binding efficiency of DENV-containing immune complexes in macrophage-like P338DI cells (Flipse et al., 2016; Gan et al., 2017).

### Feline immunodeficiency virus/simian immunodeficiency virus/human immunodeficiency virus vaccines

According to the WHO, the HIV pandemic has claimed 36.3 million lives since its discovery in the 1980s (Barré-Sinoussi et al., 1983), and 37.7 million people are currently living with HIV (HIV/AIDS, 2021). HIV infection leads to a severe acute infection in which the virus actively replicates in the lymph nodes and blood. Macrophages are thought to act as major reservoirs of HIV infection and facilitate viral dissemination throughout the body (Naif, 2013). HIV infections are accompanied by the massive destruction of CD4^+^ T cells, followed by a gradual decay in immune system regulation and responsiveness. This subsequently leads to slow progression towards AIDS for the vast majority of HIV-infected individuals (Picker and Watkins, 2005; Naif, 2013). The main receptor for HIV is thought to be the CD4 protein (Douek et al., 2002) (which explains its massive entry into CD4^+^ T cells) and cell entry usually requires binding to co-receptors, mainly the chemokine receptors CCR5 and/or CXCR4 (Moore et al., 2004; Naif, 2013). Entry into target cells is mediated by the envelope protein, composed of trimers of gp120 and gp41 subunits, involved in receptor binding and membrane fusion, respectively (Wilen et al., 2012).

Despite the existence of efficient anti-retroviral therapy (INSIGHT START Study Group et al., 2015), there are still many obstacles to achieving a cure for HIV with complete remission (Pitman et al., 2018). The HIV pandemic continues to spread and the development of efficient vaccines, as difficult and demanding as it has been up to the present, appears to be crucial. Of note, anti-HIV vaccines have been primarily designed as prophylactic treatments but candidate therapeutic vaccines with the aim of boosting immune responses in infected patients are gaining interest (Pitman et al., 2018). Despite many attempts (Huisman et al., 2009), several obstacles have been encountered that prevents the development of a successful vaccine against what is considered by many to be one of the most challenging pathogens known to date. The observation that vaccine candidates can lead to VAED in certain preclinical and clinical models is one of them.

The earliest report of disease enhancement post-vaccination and challenge with this type of virus dates back to 1992 and involved cats vaccinated against the feline immunodeficiency virus (FIV) with different ISCOM-based vaccines (Hosie et al., 1992). Despite the development of neutralizing antibodies in some groups, the vaccinated cats were not protected from infection, and all became viremic earlier than that in the unvaccinated control, thus highlighting VAED through mechanisms other than the simple lack of the neutralizing ability of antibodies (Hosie et al., 1992). In further studies, the vaccination of cats with various vaccines including the FIV envelope led to accelerated viremia and enhancement of infectivity. Although the enhancement was again transferable to previously-naïve cats by plasma transfer (Siebelink et al., 1995; Osterhaus et al., 1996), the correlation with the presence or absence of neutralizing antibodies has never been clearly established (Richardson et al., 1997)(Karlas et al., 1999; Giannecchini et al., 2002). Of note, other than pre-existing vaccine-mediated humoral responses, it was suggested that an increase in FIV-target cells following immunization could also partially contribute to enhanced cell-mediated pathogenesis due to increased opportunities for viral replication in the early phases of infection (Richardson et al., 2002). This included the expansion of specific CD4^+^ T cells (Schwartz, 1994), CD134^+^ cells (Willett et al., 2006), which is the primary receptor for FIV (Shimojima et al., 2004; Reggeti et al., 2008), and CXCR4^+^ cells (de Parseval et al., 2004).

As hypothesized for a number of anti-FIV vaccines in cats, a mechanism of lymphocyte activation-mediated enhancement of disease has been suggested for anti-simian immunodeficiency virus (SIV) vaccines, in which vaccine-mediated priming of CD4^+^ T cells against a CD4^+^ T cell-tropic virus (Douek et al., 2002) led to enhanced viral replication. Rhesus macaques vaccinated with a chimeric DNA vaccine showed only partial control of viral replication after the SIV challenge relative to controls. Vaccinated macaques showed SIV-specific CD4^+^ T cells, as well as IFNγ-producing CD8^+^ T cells, in the early phases of infection. However, control over viremia failed to be maintained by a number of animals during the chronic phase of infection, despite the preservation of IFNγ-producing CD8^+^ T-cell levels, resulting in increased viral loads, the loss of CD4^+^ T cells, and rapid progression to AIDS (Lun et al., 2004). Thus, although the decline in SIV-specific CD4^+^ T-cell numbers was not explained, it was speculated that these cells may have had a role in supporting SIV replication (Lun et al., 2004). This has been confirmed in another study with a vaccine candidate which induced non-neutralizing anti-SIV envelope antibodies and weak anti-SIV CD8^+^ T-cell responses. After the heterologous SIV challenge, vaccinated macaques showed greater and earlier viremia than control macaques, as well as a faster decline in CD4^+^ T cell number and accelerated AIDS progression (Staprans et al., 2004). In this approach, SIV-specific CD8^+^ T-cell levels were inadequate, whereas CD4^+^ T-cell levels increased early after the challenge, subsequently correlating with enhanced SIV replication. SIV-specific CD4^+^ T cells were shown to be proliferating memory cells that were primed by vaccination and rapidly mobilized upon infection (Staprans et al., 2004).

Over the years, many attempts have been made to develop anti-HIV vaccines, most of which resulted in the induction of broadly neutralizing antibodies (bNAbs) as an overarching goal. Although most candidates were removed from the clinical pipeline due to poor efficacy (Sahay et al., 2017; Pitman et al., 2018), the adenovirus type 5 (Ad5)-vectored vaccine expressing HIV gag/pol/nef proteins, a vaccine aimed to induce cell-mediated immunity, was halted due to the risk of VAED. The vaccine initially showed promising results in preclinical evaluations in NHP models in terms of attenuating the infection in the acute phase, the maintenance and recovery of CD4^+^ T cells, and the control of viremia during the chronic phase of infection (Shiver et al., 2002). A phase IIb test-of-concept randomized double-blinded clinical trial (STEP) was conducted in high-risk groups in North America, South America, the Caribbean, and Australia to investigate the prevention of HIV acquisition and/or reduction of viral load post-HIV infection. Preliminary results showed no efficacy and higher rates of infection in the vaccinated group (27% of recipients) compared to the control group (3% of recipients) (Cohen, 2007; Buchbinder et al., 2008; McElrath et al., 2008; Moore et al., 2008; Duerr et al., 2012). Although enhanced replication of SIV in NHP models mainly supported a pathogenic role for anti-envelope antibodies (Siebelink et al., 1995; Osterhaus et al., 1996), it is unlikely that this mechanism is involved here, as the *env* gene was absent from the adenovirus vector construct. Although none were truly validated, several factors, most of them not associated with HIV-specific immune responses, were hypothesized to be associated with this observation (Sahay et al., 2017). One factor was prior humoral immunity to the Ad5 vector. A higher number of infections occurred among recipients that had elevated titers of pre-existing Ad5-specific antibodies, with a 2- to 3-fold higher rate of infections among vaccinated patients than in the placebo group. No differences were observed in terms of increased susceptibility to infection between the two groups among recipients with no pre-existing immunity to Ad5. This dose-effect relationship may indicate, but does not prove, the correlation between prior immunity to the Ad5 virus and susceptibility to HIV infection. In preclinical evaluation, the SIV-Ad5 prototype construct was used with various vaccine candidates and led to the control of viremia, with no evidence of VAED (Shiver et al., 2002; Casimiro et al., 2005; Liang et al., 2005; Wilson et al., 2006). In addition to the circumcision status and sexual behaviors of males involved in the clinical trial (Koblin et al., 2012), both “immune capacity” and host genetics were suggested to have played a role in the outcome of the clinical trial (Moore et al., 2008). Furthermore, the non-specific release of IFNγby CD4^+^ T cells, rather than HIV-specific responses, was also suspected (Huang et al., 2014).

Although the study outcomes may never be fully deciphered, the STEP study raised fundamental scientific and ethical questions that need to be addressed before moving forward in the quest for an anti-HIV vaccine, and the extent to which vaccination induces cellular immunity could be the answer. The pathological mechanisms that lead to AIDS and host-pathogen interactions (Überla, 2008) continue to be studied to better understand how to defeat what is considered to be one of the most challenging pathogens known to date. Many promising strategies are being evaluated worldwide (Barouch and Korber, 2010; Burton, 2019).

### SARS-CoV-1 and MERS-CoV vaccines

Coronaviruses have been known to infect a wide range of species, with low-pathogenic coronaviruses infecting the upper-respiratory tract, commonly causing seasonal cold-like respiratory diseases in humans. There are other highly pathogenic strains that pose a considerable threat to public health. They infect the lower-respiratory tract and induce a hyper-inflammatory disease, potentially leading to pneumonia and, in the most severe cases, acute lung injury (ALI) and acute respiratory distress syndrome (ARDS) (Channappanavar and Perlman, 2017). The first epidemic was recorded in 2002-2003 with SARS-CoV-1. It started in in the southeast of China before reaching a total of 29 countries (WHO, 2002) and infecting 8,000 people (Peiris et al., 2004). The mortality rate was approximately 10%, reaching up to 50% in the elderly (SARS, 2003). A second epidemic took place in 2012-2013 in the Middle East, where MERS-CoV infected people in a total of 27 countries and caused 858 known deaths (MERS-CoV, 2019). Infection with SARS-CoV-1 is initiated by binding of the receptor binding domain (RBD) of the Spike protein to the angiotensin-converting enzyme 2 (ACE2) receptor on host cells (Du et al., 2009; Li W. et al., 2003; Davidson et al., 2020), followed by viral fusion via the heptad repeat 2 (HR2) domain (Du et al., 2009). Of note, the ACE2 protein is expressed in many tissues (Davidson et al., 2020), including in the immune system, mainly by monocytes and macrophages, making them targets for infection (Nicholls et al., 2003; Boumaza et al., 2021; Junqueira et al., 2022). Following natural infection with SARS-CoV-1, anti-Spike and anti-nucleocapsid (N) antibodies appear to be the dominant humoral response in serum (Chang et al., 2004; Xu and Gao, 2004; Du et al., 2009), along with cellular responses (Xu and Gao, 2004; Du et al., 2009). They are predictive of disease outcome early following infection (Zhang et al., 2006) and persist for a long time (Li et al., 2006). Due to its role in viral attachment and entry, and as one of the main targets of antibodies, the Spike protein is the main target for anti-SARS vaccines and treatments (Du et al., 2009). Although many vaccine candidates, using different technologies, were initiated, safety alerts were reported after the observation of potential VAED both *in vitro* and *in vivo*.

*In vitro*, SARS-CoV-1 has been found to infect and replicate circulating immune cells in the absence of ACE2 (Li L. et al., 2003). In a study using ACE2 and FcRII-bearing HL-CZ human promonocyte cells, anti-Spike antibodies were associated both with efficient neutralization or ADE in a concentration-dependent manner; sera with diluted anti-Spike-antibodies tended to enhance infection, whereas that with concentrated anti-Spike-antibodies promoted protection through neutralization (Wang et al., 2014). Immunization of mice and golden Syrian hamsters with a full-length recombinant trimeric Spike protein (triSpike) elicited strong, efficient, and long-lasting neutralizing immune responses against SARS-CoV-1. However, they were also shown to promote viral entry into human B cells using FcγRII-dependent and ACE2-independent pathways, but not mouse macrophages, despite the presence of these FcRs (Kam et al., 2007). The sera of mice immunized with a recombinant SARS-CoV-1 Spike protein adjuvanted-vaccine showed antibody-dependent infection of human macrophages via the same mechanism (Yip et al., 2014). However, it was demonstrated that human macrophages do not support productive replication. Jaume et al. also showed non-productive infection of SARS-CoV-1 in *in vitro* models, (Jaume et al., 2011). Indeed, cooperation between phagocytic cells (especially monocyte-derived infiltrating macrophages and alveolar macrophages) and anti-viral antibodies have been found to be essential for the clearance of SARS-CoV-1 in mouse models (Yasui et al., 2014).

The mouse models of SARS-CoV-1 infection have shown that several types of vaccine can induce the exacerbation of eosinophilia and the induction of Th2-mediated inflammatory pulmonary pathology independently of protection (Bolles et al., 2011; Tseng et al., 2012). The formulation of recombinant Spike protein vaccines with an alum adjuvant was associated with decreased immunopathology (Tseng et al., 2012; Chen W. H. et al., 2020). Of note, the protective role of vaccine-induced memory CD8 T cells, both in terms of immunity and preventing immunopathology, was highlighted in the mouse model following vaccination with a recombinant vaccinia virus (Channappanavar et al., 2014). Nevertheless, vaccinating macaques with a recombinant MVA virus encoding full-length SARS-CoV-1 glycoprotein (ADS-MVA) induced infiltration of the lungs by monocytes and macrophages and, overall, enhanced Spike-specific ALI (Liu et al., 2019). Anti-Spike antibodies may promote phenotypic and functional alterations of wound-healing macrophages to become pro-inflammatory, without acting on already inflammatory macrophages, leading to unrestrained inflammation and lung damage (Liu et al., 2019). Importantly, this happened despite the presence of high titers of neutralizing antibodies. FcγR-blockade reduced this process by diminishing the production of pro-inflammatory cytokines (Liu et al., 2019). Increased levels of IL-8 and IL-6 (Liu et al., 2019) also suggest a potential role of Th17-associated cytokines in SARS-CoV-1 vaccine-mediated immunopathology (Hotez et al., 2020). Moreover, Th17 is involved in eosinophil infiltration and activation (Bolles et al., 2011). Furthermore, several studies highlighted decreased pathology when vaccines (subunit or whole-inactivated) were associated with an insulin-based adjuvant (Honda-Okubo et al., 2015). Wang et al. immunized rhesus macaques with various peptide-based vaccines containing B cell-epitopes of the Spike protein (Wang et al., 2016) identified from the plasma of convalescence SARS patients. Immunization with some epitopes successfully protected the animals from infection post-challenge, whereas immunization with others lead to enhanced pathology. This study highlights a convenient strategy that should be considered for the development of safe vaccines, which consists of selecting protective epitopes for immunization and removing those that may induce VAED (Wang et al., 2016). Importantly, the authors produced monoclonal antibodies against these immunodominant epitopes and showed, *in vitro* in Vero E6 cells, which lack FcRs, that antibodies against “protective” epitopes block viral entry, whereas two antibodies against the “pathogenic” epitopes elicited enhanced infection (Wang et al., 2016). Nonetheless, the histopathological data published by Tseng et al. (2012); Liu et al. (2019), and Wang et al. (2016) have been criticized due to their poor quality, difficulties in interpretation, and the absence of a histopathological scoring system, which may have biased the interpretation (reviewed in Gartlan et al. (2022)). In contrast to previous results, although protective neutralizing immune responses were elicited (Zhou et al., 2005), Luo et al. showed that immunization of rhesus macaques with an inactivated SARS-CoV-1 vaccine led to protection rather than enhanced infection, even in the context of low levels of neutralizing antibodies (Luo et al., 2018). Qin et al. showed similar results with another inactivated vaccine in cynomolgus macaques (Qin et al., 2006).

Concerning MERS-CoV, immunization of mice with a whole-inactivated virus vaccine, either adjuvanted or non-adjuvanted, led to increased infiltration of the lungs by eosinophils, elevated IL-15 and IL-13 levels, and overall lung injury (Agrawal et al., 2016). However, the MERS-CoV challenge of NHPs following vaccination with a DNA vaccine (Wang et al., 2015) or Spike RBD domain subunit vaccine (Lan et al., 2015; Wang et al., 2015) with the RIBI adjuvant did not lead to immunopathology or VAED.

## Have SARS-CoV-2 vaccines and vaccine candidates shown any risks of VAED?

Severe acute respiratory syndrome-coronavirus 2 emerged from Wuhan (China) at the end of 2019 and led to the ongoing COVID-19 pandemic, which was declared by the WHO in March 2020 after having spread to more than 110 countries. As of the end of April 2022, more than 512 million COVID-19 cases had been registered worldwide, with nearly 6.13 million deaths (Worldometer, 2022). The emergence of a number of variants-of-concern after a long period of genetic stability has led to numerous rebounds of the pandemic, both in terms of altered transmission and mortality rates, as well as in terms of sanitary restrictions (Acuti Martellucci et al., 2020; Fontanet et al., 2021). The case fatality rate depends on the period (and hence the variant in question and the sanitary restrictions put in place) and localization, as well as the age and risk factors of individuals. Although the host-pathogen interactions are still being investigated, much remains to be discovered to fully understand the pathogenesis associated with the infection. Two receptors, ACE2 and TMPRSS2, have been shown to enable viral entry into target cells (Davidson et al., 2020; Beyerstedt et al., 2021; Koch et al., 2021). Infection with SARS-CoV-2 can lead to a wide spectrum of clinical manifestations. Most people experience an asymptomatic form of infection, whereas some require hospitalization after developing the severe pulmonary disease (Xu et al., 2020) with acute respiratory distress syndrome (ARDS). Over-activation of the immune system, with predominant local inflammation in the alveolar space (Martines et al., 2020), followed by systemic inflammation and the development of a cytokine storm is thought to be responsible for severe cases (Gao Y. et al., 2020; Huang et al., 2020; Jafarzadeh et al., 2020; Ragab et al., 2020; Wang et al., 2020). Aberrant activation and accumulation of macrophages in tissues are thought to account for the bulk of inflammation (Wang et al., 2020). There may also be a positive feedback circuit between infected macrophages and T cells in the contribution to alveolar inflammation and pneumonia (Grant et al., 2021).

Within a few months, dozens of vaccines entered the preclinical and clinical development pipeline, with many different technologies being evaluated (WHO, 2020), in an unprecedented race against the COVID-19 pandemic. A few were approved for an emergency, limited, and then general use within an extraordinarily short space of time due to accelerated preclinical and clinical evaluation (Graham, 2020; Haynes et al., 2020; Feraoun et al., 2021). Among other factors, an outstanding international coalition, enormous funding from both the private and public sectors, tremendous advances in technology, and previous research on SARS-CoV-1 and MERS-CoV made this incredible achievement possible. As of the end of April 2022, several vaccines against SARS-CoV-2 had been approved, with more than 150 have already reached the clinical phase and another 200 under pre-clinical evaluation (WHO, 2020; Covid-19 Vaccine, 2022). These vaccine candidates and approved vaccines are based on different platforms and formulations, but most target the Spike protein (WHO, 2020; Covid-19 Vaccine, 2022).

There were initial concerns about the possibility that the new SARS-Cov-2 vaccines may carry a risk of VAERD (Arvin et al., 2020; Halstead, 2021; Jamrozik et al., 2021a; Munoz et al., 2021; Gartlan et al., 2022), but two years of epidemiological data in clinical trials and widespread administration of these vaccines in the general population have made it possible to alleviate them. These concerns were based on several observations, a number of which are mentioned below:

A) The genetic proximity between SARS-CoV-2 and the other pandemic coronaviruses (SARS-CoV-1, MERS-CoV) for which VAED has been described.
B) As for vaccines against other pandemic coronaviruses, the production of anti-Spike antibodies, which was partially associated with VAED in a few studies, is also the main aim of many vaccines against SARS-CoV-2.
C) Early suspicions arose that the induction of high antibody titers the following infection with SARS-CoV-1 and SARS-CoV-2 may be associated with a worse clinical outcome (Hsueh et al., 2003; Zhao et al., 2020).
D) The lack of cross-neutralization of antibodies in serum from patients infected with SARS-CoV-1 against SARS-CoV-2 (Anderson et al., 2020). However, Tan et al. demonstrated that immunization of SARS survivors with the BNT162b2 mRNA vaccine (Pfizer – BioNTech vaccine (Polack et al., 2020)) induced a broad spectrum of pan-sarbecovirus neutralizing antibodies in humans (Tan et al., 2021). The ability to generate such cross-neutralizing antibodies with vaccines was already shown in macaques (Saunders et al., 2021) and mice (Martinez et al., 2021).
E) Antibodies that enhance infection in cellular models *in vitro* have been isolated from SARS-CoV-1 and SARS-CoV-2 patients, although these did not cause immunopathology *in vivo* in NHP and mouse models (Li et al., 2021) and did not lead to aberrant cytokine production (Maemura et al., 2021).
F) Phagocytic cells, such as monocytes and monocyte-derived macrophages (MDMs), are susceptible to SARS-CoV-2 infection, both *in vitro* and *in vivo*, through antibody-mediated pathways (Wan et al., 2020). These immune cells were indeed shown to be permissive to infection, but not to viral replication, meaning that the replication, production, and dissemination of infectious particles do not take place (Wang et al., 2020; Yang D. et al., 2020; Zheng et al., 2021; Junqueira et al., 2022). However, although abortive, the infection of monocytes and macrophages causes aberrant cytokine production (the “cytokine storm”) and massive lung and subsequent systemic inflammation, which may contribute to the overall pathogenesis and severity of COVID-19 (Wang et al., 2020; Yang D. et al., 2020; Zheng et al., 2021; Junqueira et al., 2022). Indeed, in a humanized-mouse model, SARS-CoV-2-infected macrophages are involved in the inflammatory pathogenesis of COVID-19 through the activation of inflammasomes and the induction of pyroptosis (Sefik et al., 2022b). Other groups have suggested that although productive replication in these cells does not take place, infectivity is preserved and they can disseminate throughout the body and act as “trojan horses” by enabling the virus to invade surrounding replication-permissive cells (Percivalle et al., 2021). Such transmission was shown to be hindered by human neutralizing antibodies and anti-Spike monoclonal antibodies (Percivalle et al., 2021). Although these observations were all made in the context of antibodies generated through natural infection, Junqueira et al. suggested that virus uptake and, hence, disease severity, did not increase in the presence of plasma from mRNA-vaccinated recipients (Junqueira et al., 2022).

There is a substantial body of preclinical and clinical data that highlights the benefits of anti-SARS-CoV-2 vaccination relative to the risk of enhanced disease. Indeed, many anti-SARS-CoV-2 candidate vaccines have been tested in NHP models and, contrary to what was observed for SARS-CoV-1 and MERS-CoV, no evidence of VAED has been observed. Protection generally correlates with high titers of species-specific neutralizing antibodies. Overall, no lung-associated immunopathology or signs of VAERD have been described after the challenge of various animal models with SARS-CoV-2 subsequent to vaccination with an mRNA vaccine (Corbett et al., 2020), recombinant adenovirus vaccine (Chen Y. et al., 2020), recombinant Spike protein vaccine (Yang J. et al., 2020), two-component Spike nanoparticle vaccine (Brouwer et al., 2021), adjuvanted-subunit vaccine (Tian et al., 2021), subunit vaccine targeting CD40-expressing APCs (Marlin, 2021), or DNA vaccine (Yu et al., 2020). Safety in animal models has also been reported with different inactivated vaccines. The PiCoVacc inactivated vaccine-induced neutralizing antibodies in mice, rats, and rhesus macaque models and overall protective immunity against SARS-CoV-2 challenge in rhesus macaques (Gao Q. et al., 2020). No ADE was observed, even in the animals that received a medium dose of vaccine and produced only limited titers of neutralizing antibodies (Gao Q. et al., 2020). Bewley et al. challenged ferrets and NHPs with SARS-CoV-2 following vaccination with a formalin-inactivated vaccine (Bewley et al., 2021). Among ferrets, two animals showed a transient slight enhanced-disease by day 7, characterized by bronchiolitis, eosinophilic infiltrate, and perivascular cuffing. This phenomenon was not observed in macaques, in which vaccinated animals were protected from the lung pathology associated with SARS-CoV-2 infection relative to control non-vaccinated groups (Bewley et al., 2021). Yoshikawa et al. have developed a lethal model using BALB/c mice that can be used to provide new insights on the potential occurrence of VAED following mouse-passaged SARS-CoV-2 infection (Iwata-Yoshikawa et al., 2022). This model could be a practical way for the preclinical evaluation of vaccines. The authors obtained Th1- or Th2-shifted immune responses with adjuvant-driven vaccines. Similar to mouse models of SARS-CoV-1 and MERS-CoV, they showed that an imbalance in Th1 and Th2 responses (Th1 < Th2) contributes to transient immunopathology mediated by eosinophil infiltrates in the pulmonary tract after challenge with mouse-adapted SARS-CoV-2. However, the level of neutralizing antibodies had no impact on the severity of the disease.

Another global concern is the emergence and spread of variants and the cross-protection conferred by the anti-SARS-CoV-2 vaccines, which target the initial/prototypic “Wuhan strain” (Hacisuleyman et al., 2021; Naaber et al., 2021; Ricke, 2021; Thomas et al., 2021; Covid World Vaccination Tracker, 2022). NHPs were vaccinated with three doses of SARS-CoV-2 inactivated vaccine and then challenged with different variants (prototypic SARS-CoV-2, Alpha (B.1.1.7), Beta (B.1.351), Delta (B.1.617.2), and Omicron (B.1.1.592 variant) (Krause et al., 2021). The vaccinated animals experienced a reduction in viral shedding and replication, as well as improved pathology in the lungs, relative to unvaccinated control animals inoculated with the SARS-CoV-2 variants. Importantly, they developed neutralizing antibodies against the variants, hence demonstrating cross-protection and the absence of VAED. Limitations of this study included the lack of statistical analysis due to the small number of animals in each group. The fact that the antibodies elicited by vaccination against the initial Wuhan strain are cross-protective against SARS-CoV-2 variants and are not associated with a risk of VEAD has also been demonstrated in the clinic (Arora et al., 2021; Cele et al., 2021; Karim, 2021; Reynolds et al., 2022; Xie et al., 2022).

Hence, contrary to what was hypothesized in the months following the marketing of SARS-Cov-2 vaccines (Mileto et al., 2021), there is today overwhelming evidence to reasonably disregard any potential risk of VAED associated with anti-SARS-CoV-2 vaccine-elicited immune responses. Millions of doses of vaccines, based on different immunization strategies, have been administered in the past months (Wolz et al., 2022) without reports of VAED. Finally, no epidemiological or clinical evidence has been documented that suggests that the waning of vaccine-induced antibodies, which has been observed in the months following vaccination (Groß et al., 2022; Nemet et al., 2022), might promote enhanced-pathology.

## Discussion

Although occurring rarely, VAED is still a threat to vaccine development, and, by extension, to public health as a whole. Hence, there is an undeniable necessity to further assess the safety of future vaccine candidates, the extent to which they might lead to VAED, and how this can be minimized. However, scientists are faced with evident technical difficulties and knowledge gaps in this field, which highlights the continued efforts that are required to ensure the harmlessness of future vaccines.

One of the main challenges is that, despite the knowledge that VAED has occurred in certain circumstances, no biomarkers, immunological findings, or clinical manifestations have been shown to separate VAED from severe infection. In addition, the rarity of VAED, the time between vaccination and the potential occurrence of VAED, as well as the lack of a common causative mechanism, considerably impede the prediction, surveillance, and monitoring of VAED. Given the urgent need to rapidly develop safe anti-SARS-CoV-2 vaccines and the lack of a definition for VAED and the availability of specific guidelines on how it can be assessed during preclinical and clinical trials, a Brighton Collaboration VAED group was formed in March 2020. During this consensus conference, various experts in the biomedical and public health fields were brought together by the Coalition for Epidemic Preparedness Innovations (CEPI) to discuss, among other things, current knowledge concerning the assessment of VAED in the accelerated development of anti-COVID-19 vaccines (Lambert et al., 2020; Munoz et al., 2021). A definition for VAED was established, as well as defined levels of certainty for VAED evaluation (Munoz et al., 2021).

To reduce the knowledge gap and better characterize VAED, efforts must be primarily made to better understand the immune mechanisms associated with vaccine-mediated protection and pathology. For example, the yellow fever vaccine is considered to be one of the safest and most-effective vaccines ever developed, but despite having been used for decades, the mechanisms involved in its success are not known and studies are now accumulating to decipher them (Querec et al., 2009; Wieten et al., 2016; Staples et al., 2020). In the same way that a growing number of scientists are focusing on describing immune signatures and early biomarkers associated with protective immunity following vaccination (Van Tilbeurgh et al., 2021), those potentially associated with pathogenic immune responses need to also be brought to light. A Vaccines and Related Biological Products Advisory Committee (VRBPAC) meeting took place in 2017 to discuss the immunopathogenesis of VAED and the preclinical and clinical considerations and data required to support the development of vaccines intended for RSV-naïve children (Browne et al., 2020). It was established that certain interdependent responses might be predictive of VAED or at least lead to the suspicion of VAERD; namely, dominant Th2 immune responses, lung eosinophilia, high titers of low-avidity/non-neutralizing antibodies, and the absence of specific CD8^+^ T cells (Browne et al., 2020). Of note, the findings concerning VAED are pathogen-specific, and even epitope-specific, meaning that although the mechanisms may be somewhat similar, current knowledge does not allow extrapolation to other vaccines.

Investigation of the possibility of VAED is crucial during clinical trials for decision-making on vaccine licensing by regulatory agencies. To minimize risks for the participants, compromises have to be made between the scientific interests of the trial and the safety of the participants (Jamrozik et al., 2021a). For example, Phase III clinical trials that test for efficacy should involve a rigorous selection of participants in areas with a higher probability of encountering the virus to reduce the required time and hence the cost of the trial, but for whom the outcome of potential VAED would not be as severe. In the case of anti-RSV vaccines, it was established that clinical studies must first be conducted in a healthy adult population and RSV-experienced children so that safety is demonstrated before being tested on RSV-naïve children (Browne et al., 2020). In the majority of cases, the risk of VAED is brought to light during preclinical stages or early clinical trial phases. Nonetheless, VAED is quite infrequent and generally occurs a long time post-immunization, as it manifests when (and only if) vaccine recipients are exposed to the pathogen and infected subsequent to vaccine administration. In this sense, VAED can sometimes only be detected in the later phases of clinical trials, as was the case for the HIV-Ad5-vectored vaccine candidate (Cohen, 2007; Buchbinder et al., 2008; McElrath et al., 2008; Moore et al., 2008; Duerr et al., 2012), or even post-license, as was the case for the Dengvaxia vaccine (Hadinegoro et al., 2015; Villar et al., 2015; Sridhar et al., 2018) and the FI-MV vaccine (Fulginiti et al., 1967; Nader et al., 1968; Martin et al., 1979; Smatti et al., 2018). Hence, vigorous surveillance of VAED is necessary for the pharmacovigilance phases post-license.

One solution to reduce the time of clinical trials could be the implementation of human-challenge trials (also called controlled human infection model (CHIM) studies), which involve the intentional infection of healthy and consenting individuals in a monitored environment. CHIM studies have received increased consideration in biomedical research, given the invaluable insight they can provide in terms of host-pathogen interactions, immune correlates of protection, accelerated vaccine development, etc. (Sekhar and Kang, 2020), even more so during the COVID-19 sanitary crisis. Despite the WHO guidance (WHO, 2017), and the strict ethical frame and thorough supervision, the deliberate exposition of humans to an infectious disease poses many ethical concerns, and is highly criticized in many countries. Despite much debate due to obvious ethical issues (Baay and Neels, 2020; Edwards and Neuzil, 2022), the idea of implementing CHIM studies in the context of the ongoing COVID-19 pandemic was considered (Baay and Neels, 2020; Deming et al., 2020; Jamrozik et al., 2021b; Edwards and Neuzil, 2022) to respond to the emergency of developing safe and effective vaccines. The first trial was conducted on young adults in the UK early in February 2021 (Oxford Vaccine Group, 2020), the first results having been published recently (Killingley et al., 2022). Young and healthy adults previously immunized by natural infection or vaccination were infected with SARS-CoV-2 in a controlled manner, with no safety issues having been declared. In the context of VAED, the trial would need to be expanded to the investigation of the impact of vaccination prior to SARS-CoV-2-challenge. The main limitations are mainly the small number of participants and the fact that participants are carefully screened to ensure that they are perfectly healthy and do not have any risk factors for the disease. Indeed, due to their rareness, VAED events may not be detectable, if they exist, or if the participants are limited in numbers. In addition, the observations made on these participants may not be extrapolatable to the general population, which is much more heterogeneous and includes people with specific attributes that could influence disease severity independently of vaccination. In addition, CHIM studies to evaluate the risks of VAED may require enrolling SARS-CoV-2 naïve participants to study the effect of prior immunity to pandemic coronaviruses on VAED occurrence, as this has been an issue in the past with DENV serotypes (Halstead, 2018).

According to the definition established by the Brighton Collaboration (Munoz et al., 2021), the possibility of VAED includes an “increased frequency of severe outcomes (including severe disease, hospitalization, and mortality) when compared to a non-vaccinated population (control group or background rates)”. Notably, the large-scale clinical experience with a variety of SARS-CoV-2 vaccines in humans, both before and after licensing, has provided no evidence of breakthrough enhanced disease (AAAS, 2021) that could justify delaying or interrupting SARS-CoV-2 vaccine accessibility and administration. On the contrary, it has highlighted the beneficial role of vaccinations (Suthar et al., 2022). Naturally, in the context of VAED and given the proximity between the viruses, this observation frames an important research opportunity to understand why laboratory animals are given SARS-CoV-1 and MERS-CoV vaccines experienced more-or-less abnormal responses to a wild virus challenge, whereas the current generation of SARS-CoV-2 vaccines has not sensitized humans nor animal models.

For ethical and safety reasons, only limited studies can be conducted on humans, with a very strict framework and legislation (European Commission, 2014; FDA, 2015). Animal models are imperfect but highly useful tools to bridge *in vitro* proof-of-concept studies and human clinical trials. Although preclinical testing is not a prerequisite for clinical trials, it can unquestionably provide valuable information on the safety and immunogenicity of many vaccine candidates (Kiros et al., 2012), when appropriately designed and conducted. Animal models often provide key information to decide whether to pursue vaccine development or not, as has been the case for vaccines against numerous infectious agents, including anti-SARS-CoV-2 vaccines (Chen Y. et al., 2020; Corbett et al., 2020; Yang J. et al., 2020; Yu et al., 2020; Brouwer et al., 2021; Marlin, 2021; Tian et al., 2021; Gao Q. et al., 2020; Bewley et al., 2021; Krause et al., 2021). Such studies can be systematically initiated to focus on early events or biomarkers that may indicate the risk of VAED, or at least provide mechanistic insights on the associated pathology. Another major advantage of preclinical models is the possibility of conducting extensive longitudinal studies and the availability of a greater number of samples, in terms of both sampling frequency and sample diversity. Nonetheless, it is crucial to keep in mind that, due to physiological differences between animals and humans, studies in animal models are used to enlighten scientists on certain mechanisms but are not able to replace studies in humans (Cohen, 2006). Consequently, signs can occur in preclinical studies that raise doubts on safety parameters but that will not be observed in humans and, hence, do not predict VAED. Conversely, a vaccine without any safety concerns in animal models can still induce VAED in humans. By extension, potential mechanisms or biomarkers may be identified but they are generally not sufficiently understood to be efficiently translated into the design of the vaccine.

For optimal results, animal models need to be adequately fit-for-purpose. Indeed, each animal model comes with physiological and technical advantages and disadvantages (Kiros et al., 2012), and the most relevant for a specific question needs to be used. In addition, differences in immune responses between species need to be taken into consideration. For example, antibodies and complements can have very different properties depending on the model and, as they are the main actors in VAED, may falsely influence protective/deleterious observations made concerning VAED. On the one hand, mice are the most used: they are less expensive, easier to handle and they have well-characterized immune responses and defined genetic background. On the other hand, despite ethical and practical difficulties, larger animals are more biologically and immunologically relevant models to study human infections and vaccine immune responses (Kiros et al., 2012), NHPs being the most compatible models (Estes et al., 2018). Indeed, NHPs reproduce human disease after infection with most human pathogens or closely-related ones, and they have similar immune responses. This is crucial to be able to evaluate the efficacy of a vaccine, but also to investigate risks for VAED since detection and monitoring of its occurrence appears challenging if the model does not display symptoms. NHP models are at the forefront of efforts to study HIV transmission and prevention, as well as SARS-CoV-2 (Chen Y. et al., 2020; Yu et al., 2020; Brouwer et al., 2021; Marlin, 2021; Tian et al., 2021) and many other infectious diseases (reviewed in Estes et al. (2018)).

When modeling on NHPs is not an option, scientists have turned to the use of more sophisticated models to reduce the lack of translatability from animal to human, examples of which include surrogate models. These are “artificial models” which can be infected with pathogens of interest only under experimental conditions (Kiros et al., 2012). The use of transgenic and knockout mouse models is highly relevant to studying the role of a particular individual gene in specific immune responses. The design of single and double knockout mice has made it possible to understand the role of IL-5 and eotaxin in the recruitment of eosinophils, which appears to be a hallmark of VAED of FI-RSV and FI-MV vaccines (Su et al., 2015). “Humanized mouse models”, in which specific human cells or tissues are engrafted (Allen et al., 2019), have also received great interest to mimic, as closely as technically-possible, the clinical experience and enhance translatability to human biomedical research (Wiles et al., 2006; Denayer et al., 2014; Fujiwara, 2018). Mouse models with human immune systems have been developed to gain insights into the function of the immune system in health and disease, host-pathogen interactions, prophylactic and therapeutic treatments against infectious diseases, including SARS-CoV-2 (Dinnon et al., 2020; Charles River, 2021; Sefik et al., 2021, 2022a,2022b), and many other research areas (Allen et al., 2019). Challenges of such models include the complexity of set-up, the risk of graft-versus-host disease, incomplete immune functions, etc., as well as the lack of adaptability of the models, which are designed for a specific research purpose and, hence, cannot support a broad array of research (Allen et al., 2019). Literature on the use of humanized mouse models in the context of VAED is not extensive. Sefik et al. used the MISTRG6-hACE2 humanized mouse model to show that SARS-CoV-2-infected macrophages contribute to the inflammatory pathogenesis of COVID-19 through the activation of inflammasomes and the induction of pyroptosis (Sefik et al., 2022b).

Despite evident and substantial similarities between a number of animal species, mainly mammals and humans, there are also physiological and genetic differences that can prevent the translation of results obtained during *in vivo* investigations to clinical application (Feinberg and Moore, 2002; Sabroe et al., 2007; Barré-Sinoussi and Montagutelli, 2015). One way to take advantage of animal models to better address scientific questions is to design studies that take into consideration both targeted subpopulations with specific attributes and the epidemiological context. Indeed, clinical trials for vaccine development are often conducted on healthy adults and rarely include populations at risks, such as infants, pregnant women, or the elderly. Targeted studies on these populations can be performed in preclinical studies. The occurrence of VAED has mostly been detected in the younger population, as the target population of most vaccines is children. Second, in the case of VAED, the possibility of setting up reinfection models could be informative, mainly in the case of DENV, for which the observed pathology could be due to repeated exposure to the antigen, both through infection and vaccination.

Lastly, closing the gap between preclinical and clinical data should involve making preclinical setups as close to that in clinical trials as possible. Standardizing technical and biological parameters between preclinical and clinical trials, by using similar scoring systems and endpoints, for instance, could be the first step. Additionally, using the same tools and techniques to measure immune and biological parameters is necessary. The reagents and other preparations used for clinical studies fall under Good Manufacturing Practices (GMP)-standards, whereas, for cost reasons, this is not necessary for preclinical studies, which could be a source of the issue. For example, Shaw et al. used a cotton rat model of VAERD following vaccination with the FI-RSV vaccine (Shaw et al., 2013b) to show that the presence of non-viral cell-culture-derived contaminants such as bovine serum albumin drives a bulk of the inflammation following challenge with RSV, while it is not the case following natural infection of children (Shaw et al., 2013b).

The observations made *in vitro* and *in vivo* during the preclinical evaluation of vaccines are used to inform decisions that will not only involve a lot of money but also affect thousands of volunteers in subsequent clinical trials. Therefore, despite being quite limited in terms of predictive power and requiring constant improvements to be more reliable and informative, animal models as they are appreciated today, are still essential in biomedical research. Concerning VAED, they are valuable in assessing the risks carried by vaccine candidates by providing information on pathological mechanisms that need to be monitored during subsequent clinical studies in humans. Although they may not be able to dissect potential pathological mechanisms *per se*, they might at least provide early signs to be taken into consideration before evaluation in humans.

Lastly, vaccine formulation strategies may be a way forward to minimize the risk of VAED. The design (Shukla et al., 2020), type, dose, formulation, site of administration (Rosenbaum et al., 2021), and administration schedule (Palgen et al., 2021) of a vaccine have a considerable impact on the vaccine-induced immune response and subsequent protective immunity (Zimmermann and Curtis, 2019). Hence, modulating these parameters may result in altered immune responses, some of which might be less prone to causing VAED. Clearly, with the many observations brought to light by past VAED experiences, avoiding vaccine strategies that generate non-neutralizing or suboptimal concentrations of neutralizing antibodies could help prevent the occurrence of VAED, as well as favor Th1-type over Th2-type responses. Consistent with these objectives, many safe vaccines that we know today have been formulated with adjuvants, which play a role in enhancing the magnitude and durability of immune responses to vaccines through the activation of innate immunity (Awate et al., 2013). The importance of early innate immune responses in setting the scene for ensuing adaptive responses is now well known (Iwasaki and Medzhitov, 2010). Thus, the proper use of adjuvants could be an option to condition and skew vaccine-specific humoral and cellular immune responses toward those that are functional, safe, and efficient (O’Hagan et al., 2017). Finally, from a theoretical point of view, the risk of VAED is greater for whole-inactivated vaccines, which aim to elicit a humoral response. Indeed, they expose the immune system to epitopes that might constitute non-neutralizing targets or be presented in non-neutralizing conformations, thus inducing suboptimal immune responses that could override those that are protective. Due to their intrinsic mechanisms, live-attenuated vaccines, vectored-vaccines, and nucleic-acid and subunit vaccines carry less risk of such immunopathology. Live-attenuated vaccines mimic natural infections and, to date, have not been shown to induce VAED (Graham, 2011; Browne et al., 2020). However, these observations are still theoretical, as in practice, VAED is not restricted to inactivated vaccines and many inactivated vaccines that have been commercialized have not caused any safety issues in terms of VAED.

Overall, predicting VAED is highly challenging in practice, certainly due to the fact that the tools that we have at our disposal are not yet sufficient and/or well-enough understood to perfectly mimic the vaccine-elicited immune responses that occur in humans. This subsequently restricts our ability to prevent their occurrence in future vaccine designs. Nonetheless, we have highlighted various research areas, in terms of model translatability and vaccine design and formulation, which can be improved to better understand VAED and overcome these limitations. Furthermore, in addition to finding ways to better predict and detect VAED in future vaccines, transparency and communication concerning VAED need to be improved by health policies to encourage public trust in vaccines.

## Author contributions

JB: analysis and writing with the guidance of RL, PM, and FM. JB, RL, PM, and FM: conceptualization and reviewing, and editing. All authors contributed to the article and approved the submitted version.

## Abbreviations

ACE2: angiotensin-converting enzyme 2
ADE: antibodydependent enhancement
Ad5: adenovirus type 5
AIDS: acute immunodeficiency syndrome
ALI: acute lung injury
COVID-19: coronavirus-induced disease 2019
CR: complement receptor
DENV: Dengue Virus
DHF: dengue hemorrhagic fever
DSS: dengue shock syndrome
FcR: Fc receptor
FI: formalin-inactivated
FIV: feline immunodeficiency virus
HIV-1: human immunodeficiency virus 1
MERS-CoV: Middle-Eastern respiratory syndrome coronavirus
MV: measles virus
NHP: non-human primate
RSV: respiratory syncytial virus
SARS-CoV: severe acute respiratory syndrome coronavirus 1
SARSCoV-2: severe acute respiratory syndrome coronavirus 2
SIV: simian immunodeficiency virus
VAED: vaccine-associated enhanced disease.
